# Infectious aetiologies of severe acute chest syndrome in sickle-cell adult patients, combining conventional microbiological tests and respiratory multiplex PCR

**DOI:** 10.1038/s41598-021-84163-3

**Published:** 2021-03-01

**Authors:** Julien Lopinto, Alexandre Elabbadi, Aude Gibelin, Guillaume Voiriot, Muriel Fartoukh

**Affiliations:** 1 Assistance Publique–Hôpitaux de Paris, Service de Médecine intensive réanimation, Hôpital Tenon, 4, rue de la Chine, 75020 Paris, France; 2 Sorbonne Université, UFR Médecine, Paris, France

**Keywords:** Sickle cell disease, Viral infection, Bacterial infection, Microbiology, Medical research

## Abstract

Acute chest syndrome (ACS) is the most serious complication of sickle cell disease. The pathophysiology of ACS may involve lower respiratory tract infection (LRTI), alveolar hypoventilation and atelectasis, bone infarcts-driven fat embolism, and in situ pulmonary artery thrombosis. One of the most challenging issues for the physicians is to diagnose LRTI as the cause of ACS. The use of a respiratory multiplex PCR (mPCR) for the diagnosis of LRTI has not been assessed in sickle-cell adult patients with ACS. To describe the spectrum of infectious aetiologies of severe ACS, using a diagnostic approach combining conventional tests and mPCR. A non-interventional monocenter prospective study involving all the consecutive sickle-cell adult patients with ACS admitted to the intensive care unit (ICU). Microbiological investigation included conventional tests and a nasopharyngeal swab for mPCR. Altogether, 36 patients were enrolled, of whom 30 (83%) had complete microbiological investigations. A bacterial microorganism, mostly *Staphylococcus aureus* (n = 8), was identified in 11 patients. There was no pneumonia-associated intracellular bacterial pathogen. A respiratory virus was identified in six patients. Using both conventional tests and nasopharyngeal mPCR, a microbiological documentation was obtained in half of adult ACS patients admitted to the ICU. Pyogenic bacteria, especially *S. aureus*, predominated.

## Introduction

Sickle cell disease (SCD) is one of the most common monogenic disorders in the world. Acute chest syndrome (ACS) is a frequent and serious complication of SCD, accounting for 70% of intensive care unit  (ICU) admissions, and being the major cause of death in adults^1,2^. ACS usually develops in 10 to 20% of patients hospitalized for a vaso-occlusive crisis (VOC), after 2.5 days of hospitalization. The syndrome is characterized by a complex pathophysiology that may involve lower respiratory tract infection (LRTI), alveolar hypoventilation and atelectasis, bone infarcts-driven fat embolism, and in situ pulmonary artery thrombosis. The clinical diagnosis of ACS includes the combination of clinical symptoms or signs, e.g. chest pain, fever, or dyspnea usually characterized by a rapid shallow breathing, with a new pulmonary infiltrate, typically lower lobe consolidation in adult patients^3^.

One of the most challenging issues for the physicians in charge of SCD adult patients with ACS is to diagnose LRTI as the cause of ACS. As a consequence, most patients with ACS are receiving antimicrobial treatment, especially when admitted to the ICU, and current practice includes routine administration of antibiotics for 7 to 10 days. However, this strategy has been rarely evaluated in adult patients^4^. In the study of Vichinsky et al. involving 671 episodes of ACS in 538 children and adults with SCD, a microbiological documentation was obtained in 38% of the cases, using conventional diagnostic tests. *Chlamydia pneumophila* and *Mycoplasma pneumonia* (30% of cases), and to a lesser degree *Staphylococcus aureus* (5%) and *Streptococcus pneumoniae* (4%) were the most frequently identified microorganisms in adult patients^5^. Syndromic respiratory multiplex PCR systems (mPCR) have been recently developed to identify targeted microorganisms involved in major respiratory infectious syndromes, using novel rapid nucleic acid amplification techniques. The usefulness of respiratory mPCR has been assessed in a paediatric cohort of sickle-cell young children experiencing ACS in whom a low rate of intracellular microorganisms was reported^6^, suggesting that a better knowledge of the microbial epidemiology was necessary, especially regarding the respective role of pyogenic bacteria, intracellular bacteria, and viruses.

We hypothesize that the optimization of the microbiological documentation of ACS might improve the use of antimicrobial drugs in sickle-cell adult patients. The objectives of this pilot study were to describe the microbial spectrum and antibiotic use in sickle-cell adult patients with ACS admitted to the ICU, using molecular diagnostic testing.

## Patients and methods

The multiplex PCR in acute chest syndrome (multipl_ACS) study was a prospective non-interventional monocenter study conducted in the ICU of Tenon Hospital, Paris, France, a tertiary University hospital and referral center for SCD. All sickle-cell adult (≥ 18 years old) patients admitted to our unit from May 2017 to May 2018 for ACS were eligible. Pregnant or lactating women, patients referred for a postoperative monitoring, and patients deprived of liberty or under legal protection measure were not included.

ACS was diagnosed based on clinical definition included in French guidelines^7^. Acute respiratory failure was defined by the presence of at least one criterion among a respiratory rate > 30 breaths/min, evidence of increased work of breathing or laboured breathing. Bedside Chest-X-Ray (C-X-R) were interpreted by the attending physicians, as part of their routine daily clinical evaluation. Lung opacities were characterized according to the Fleischner Society Glossary of terms for Thoracic imaging, defining patterns of lung parenchyma involvement (normal, consolidation, ground glass opacity) for each four radiological quadrants^8^.

The therapeutic management of ACS included bed rest, supplemental oxygen, intravenous hydration, folate supplementation, analgesics and blood products transfusion, as needed^7,9^. Whenever possible, a complete conventional microbiological work-up was performed before any antimicrobial treatment was administered, including respiratory tract specimen samples, blood cultures, and urinary antigen tests for *Legionella pneumophila* and *Streptococcus pneumoniae.* Obtaining sputum was attempted from every individual, using physiotherapy techniques and sputum induction if required. The bacterial examination of sputum included the following: assessment of the quality of sputum specimens based on Murray-Washington criteria (good-quality specimens were those with ≤ 10 squamous epithelium and > 25 leukocytes), microscopic examination using Gram stain, and cultures using standard media**.** Colony counts were read after 24- and 48-h culture. All the organisms recovered were identified and their susceptibility tested using standard techniques. A nasopharyngeal swab was performed in all patients for respiratory mPCR, using the FilmArray Respiratory Panel system (BioFire, Salt lake City, UT) that includes 17 respiratory viruses (coronaviruses, adenovirus, human metapneumovirus, human enterovirus/rhinovirus, respiratory syncytial virus, parainfluenza viruses and influenza viruses A and B) and three bacteria (*Chlamydophila pneumoniae, Mycoplasma pneumoniae,* and *Bordetella pertussis*). All patients received empiric antimicrobial treatment intravenously, combining a third-generation cephalosporin and spiramycin, according to the French recommendations for the treatment of severe ACS^7^. All methods were performed in accordance with the relevant guidelines and regulations.

A bacterial aetiology of ACS was diagnosed in the following cases: identification of a bacterial microorganism in the respiratory tract samples, yielding positive cultures at or above the threshold in the absence of prior antimicrobial therapy (or below the thresholds in the presence of ongoing or recently introduced antibiotics), or positivity of urinary antigen tests or blood culture. Non-bacterial ACS was defined by the absence of bacterial documentation regardless of any viral documentation.

### Statistical analysis

Continuous data were expressed as median (25th–75th percentiles) or mean (standard deviation) as appropriate, and were compared using the Mann–Whitney test (small sample size). Categorical variables, expressed as percentages, were evaluated using the Chi-square test or the Fisher’s exact test (small sample size). Viral, bacterial and mixed infectious aetiologies of ACS were described. Bacterial and non-bacterial aetiologies of ACS were compared. Statistical significance was defined as P values of less than 0.05. Data were analyzed using the Stata software (V 13.1, College Station, Texas USA).

### Ethical considerations

Informed written consent was obtained from all participants and/or their legal guardians. French authorities approved the study (Comité de Protection des Personnes-Sud Ouest et Outre mer III; number ID-RCB 2017-A00944-49).

## Results

**Figure 1:**
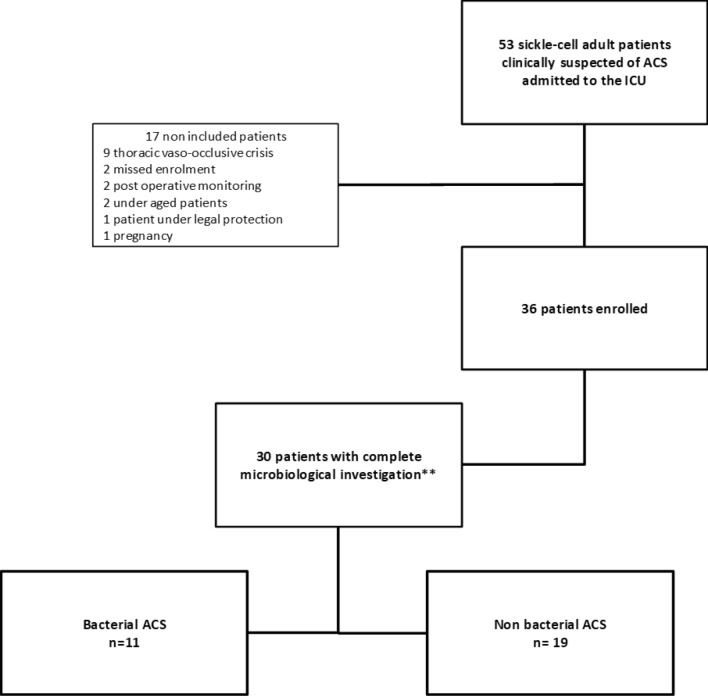
Selection of the patients.

**Figure 2:**
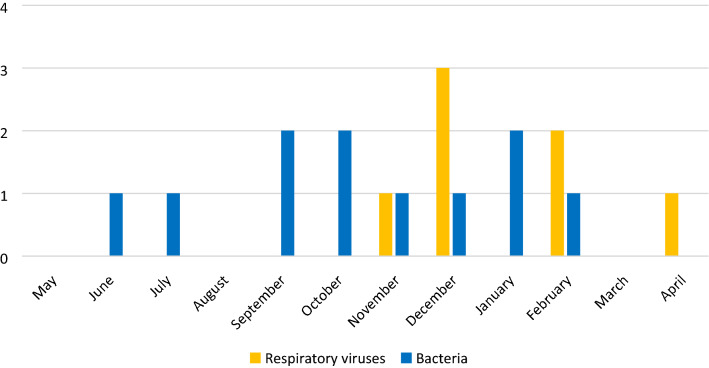
Monthly distribution of the microorganisms identified by conventional tests and mPCR.

During the study period, 53 sickle-cell adult patients clinically suspected of ACS were referred to our ICU. Of those latter, 17 were not included (postoperative monitoring, n = 2; patients under 18 years, n = 2; patient deprived of liberty, n = 1; pregnancy, n = 1; failed enrolment, n = 2 and thoracic vaso-occlusive disease, n = 9) (Fig. 1). The baseline characteristics of the 36 patients enrolled are detailed in Table 1. A complete conventional microbiological investigation was performed in 30 patients (83%), including sputum, blood cultures, and urinary antigen tests for *L. pneumophila* and *S. pneumoniae* (Table 2). Nine of these 30 patients (30%) had received antibiotics prior to sputum samplings, which were obtained after 0 [0–1] day of antibiotics administration. mPCR was performed in 31 patients (86%) (Table 2) and a respiratory virus was identified in six of them (19%), including rhinovirus (n = 3), coronavirus and influenza A co-infection (n = 1) and influenza B (n = 2). A bacterial microorganism was identified in 11 patients with complete conventional investigation (37%) (Table 2), including *Staphylococcus aureus* (all strains being susceptible to methicillin) (n = 8), *S. pneumoniae* (n = 1), *Streptococcus agalactiae* (n = 1) and *Moraxella catarrhalis* (n = 1). The monthly distribution of respiratory viruses and bacteria is shown in Fig. 2. A bacterium-virus co-infection was present in three patients: influenza B and *S. aureus,* influenza A and *S. aureus*, rhinovirus and *S. pneumoniae* (n = 1, each). Supplementary Table S1 provides detailed information on conventional microbial investigations, bacterial identification and antibiotics timing relative to sampling.

**Table 1:** Baseline characteristics of the 36 patients enrolled.

Variable	Results
**Age (years), median [25–75]**	26 [21–36]
**Sex, n (%)**	
Men	19 (53)
**BMI (kg/m**^**2**^**), median [25–75]**	21.9 [20.1–26.1]
**Sickle cell genotype, n (%)**	
SS	34 (94)
S beta Thalassemia	2 (6)
Baseline haemoglobin (g/dL), median [25–75]	8.5 [7.8–9]
**Chronic SCD complications, n (%)**	
Retinopathy	16 (44)
Pulmonary hypertension	2 (6)
Cerebrovascular disease	4 (11)
Leg ulcer	5 (14)
Biliary lithiasis	23 (64)
Kidney disease	18 (50)
Bone damage	9 (25)
**Long-term treatment, n (%)**	
Folic acid	35 (97)
Hydroxyurea	31 (86)
Transfusion/exchange transfusion	1 (3)
Erythropoietin	1 (3)
**SAPS2 on ICU admission, median [25–75]**	12 [8–17]

Results are reported as median and inter-quartile range [25–75] or number and frequency.

BMI: Body mass index; SAPS2: Simplified Acute Physiology Score 2; ICU: intensive care unit.

**Table 2:** Microbiological diagnostic work-up.

Microbiological investigations	Test performed, n	Positive result, n (%)
**Bacteriological examination of sputum**	30	11 (37)
**Urinary antigen tests**		
*Legionella pneumophila*	35	0
*Streptococcus pneumoniae*	33	0
**Blood cultures**	34	0
**Respiratory multiplex PCR**	31	6 (19)

The mPCR was not performed in 5 (14%) patients for logistical reasons. Sputum was not obtained in 6 (17%) patients, due to inability to obtain sampling of adequate cytological quality.

On ICU admission, a golden sputum (n = 8; 73% *vs.* n = 3; 16%; *P* = 0.004; Fisher exact test), dyspnoea (n = 8; 73% *vs.* n = 4; 21%; *P* = 0.009; Fisher exact test) and acute respiratory failure (n = 6; 55% *vs.* n = 2; 11%; *P* = 0.028; Fisher exact test) were more likely observed in the 11 patients with bacterial ACS than in the 19 patients with non-bacterial ACS. There were no difference in radiological patterns between both groups (alveolar consolidation in 8 patients (73%) with bacterial ACS, as compared with 14 patients (74%) with non-bacterial ACS; *P* = 1.00; Fisher exact test). Despite a similar overall duration (7 days [7–10] for bacterial ACS *vs.* 8 days [7–10] for non-bacterial ACS; *P* = 0.38; Mann Whitney test) (Table 3), the antimicrobial therapy was adjusted in all bacterial ACS patients (n = 11; 100%), including beta-lactam spectrum narrowing and spiramycin discontinuation, and in 12 (63%; *P* = 0.014, Fisher exact test) non-bacterial ACS patients (Table 3). In the latter group, macrolide therapy was discontinued (n = 7) and/or beta-lactam therapy narrowed (n = 9). There were no between-group differences in terms of initial severity of ACS, management and outcomes (Table 3).

**Table 3:** Management and outcomes of the 30 patients with ACS and complete microbiological investigations.

Outcomes	Bacterial ACS, n = 11	Non-bacterial ACS, n = 19	*P* value^a^
**ICU management, n (%)**			
Blood transfusion	5 (45)	6 (32)	0.71
Anticoagulation	3 (27)	9 (47)	0.44
High flow oxygen therapy	2 (18)	5 (26)	1.00
Invasive mechanical ventilation	0	0	–
Renal replacement therapy	0	0	–
Vasopressor support	0	0	–
**Bilateral lung injury, n (%)**	2 (18)	2 (11)	0.61
**Antimicrobial therapy**			
Overall duration (days), median [25–75]	7 [7–10]	8 [7–10]	0.38
Secondarily narrowed, n (%)	11 (100)	12 (63)	0.014
**Length of stay (days), median [25–75]**			
ICU	3 [2–7]	4 [3–5]	0.37
Hospital	11 [6–12]	8 [7–10]	0.63

ICU: intensive care unit.

^a^Results are reported as number (percentage) or median [inter-quartile range]. The corresponding between-groups comparisons were performed using the Fisher’s exact test and the Mann–Whitney test, respectively.

## Discussion

In this pilot study describing the spectrum of infectious aetiologies of ACS in sickle-cell adult patients hospitalized in the ICU, bacterial and viral microorganisms were respectively identified in 37% and 19% of cases, using a diagnostic approach combining conventional diagnostic tests and a respiratory multiplex PCR. Altogether, *S. aureus* and respiratory viruses were the most frequent microorganisms. No intracellular bacterium was identified.

The 37% incidence rate of microbial aetiologies of ACS is in accordance with that reported in previous studies^5,10^. However, the microbial spectrum differed from that described in a large series of adult and paediatric SCD patients with ACS, which reported a high proportion of intracellular bacteria, with low rates of *S. aureus* and *S. pneumoniae* (5% each)^5^. Our studied population (only adult patients), as well as the use of a sensitive diagnostic tool, may explain this discrepancy. A recent paediatric French cohort, using a similar approach, also suggested that *M. pneumoniae* was rarely involved in young children with SCD experiencing ACS^6^. Altogether, those findings raise the questions of the role of intracellular bacteria and the relevance of macrolid treatment in ACS.

In our study, the initial antimicrobial therapy was systematically narrowed in the patients diagnosed with a bacterial infection, and in two-thirds of patients with non-bacterial ACS. Of note, the initial broad-spectrum antimicrobial therapy, combining a third-generation cephalosporin and spiramycin, was maintained in 7 (37%) non-bacterial ACS patients during 8 days, which may suggest that physicians were reluctant to narrow the spectrum of antimicrobial therapy in non-bacterial ACS patients, despite the absence of bacterial species identification integrating the negative results of sputum examination (for identifying pyogenic bacteria), urinary antigen tests and mPCR (for identifying intracellular bacteria). We observed a 19% incidence rate of viral infection, with a seasonal distribution of respiratory viruses. Our findings suggest that mPCR may substantially add to the knowledge of viral and bacterial aetiologies of ACS in sickle cell adult patients. Moreover, mPCR may provide rapid results that should improve the therapeutic management of patients, by reducing unnecessary antibiotic exposure, and indicating isolation measures when respiratory viruses are identified. However, the etiologic role of a respiratory virus identified with mPCR to the contribution to a symptomatic disease in patients clinically suspected of LRTI may vary by age group and specific virus, particularly parainfluenza, coronaviruses, rhinovirus, and adenovirus in children^11,12^. In our study, we acknowledge that the asymptomatic carriage of rhinovirus may not be ruled out.

Biomarkers suggestive of bacterial infection should also be helpful for a rationale use of antibiotics. Unfortunately, we did not systematically perform blood measures of procalcitonin or CRP. A recent prospective before–after study in medical wards and ICU aimed at assessing the usefulness of procalcitonin to reduce antibiotics exposure during ACS in adult patients with SCD, and suggested that a procalcitonin-guided strategy might reduce antibiotics exposure with no apparent adverse outcomes^13^.

### Limitations

Limitations of the study are related to (i) the sample size of the population; and (ii) the probable underestimation of bacterial infection rate, due to the incompleteness of the conventional microbiological investigations in all patients, and the non-extended panel of the mPCR that did not include pyogenic bacteria. Third, blood measures of acute phase reactants suggestive of bacterial infection such as procalcitonin or CRP were not systematically performed, and should be assessed in future trials.

To summarize, a LRTI was observed in more than one-third of ACS patients who had a thorough microbiological workup, using both conventional and molecular diagnostic tests. *S. aureus* and respiratory viruses were the main microorganisms identified. No infection involving intracellular bacteria was identified. The overall duration of antimicrobial therapy did not differ according to the microbiological documentation of ACS. However, a bacterial infection led to the systematic adjustment of antimicrobial therapy. Future randomized controlled trials comparing the efficacy and safety of a pathogen-directed strategy using an extended respiratory panel mPCR, and a conventional strategy in SCD adult patients hospitalized for ACS are needed. A randomized trial combining the use of a respiratory broad panel multiplex PCR and procalcitonin to reduce antibiotics exposure in hospitalized sickle-cell adults with ACS is currently in progress (NCT03919266).

## Supplementary Information

Supplementary Information.
