# Supplementary material for: Infectious aetiologies of severe acute chest syndrome in sickle-cell adult patients, combining conventional microbiological tests and respiratory multiplex PCR
Source: Sci Rep. 2021 Mar 1;11:4837. doi: 10.1038/s41598-021-84163-3 (PMC7921101; doi:10.1038/s41598-021-84163-3)
Supplement: Supplementary file 1 — Supplementary Information. [file 41598_2021_84163_MOESM1_ESM.docx]

**Infectious aetiologies of severe acute chest syndrome in sickle-cell adult patients, combining conventional microbiological tests and respiratory multiplex PCR.**

Julien Lopinto (a,b), Alexandre Elabbadi (a), Aude Gibelin (a), Guillaume Voiriot (a,b) and Muriel Fartoukh (a,b)

(a) Assistance Publique – Hôpitaux de Paris, Service de Médecine intensive réanimation, Hôpital Tenon, 75020 Paris, France.

(b) Sorbonne Université, UFR Médecine, Paris, France

**Supplementary Table S1: Conventional microbiological investigations, bacterial identification and antibiotics timing in the 36 patients enrolled.**

| **Patient** | **Microbiological investigation**  **(P= positive test; N = negative test; NA = test not performed)** | | | | **Bacterial**  **identification** | **Antibiotics prior to**  **microbiological sampling** | **Time between antibiotics**  **and sampling (days)** |
| --- | --- | --- | --- | --- | --- | --- | --- |
|  | Respiratory tract sample | Blood culture | Urinary antigen test for *Legionella pneumophila* | Urinary antigen test for *Streptococcus pneumoniae* |  |  |  |
| **No 1** | *NA* | N | N | *NA* | - | no | 0 |
| **No 2** | *NA* | N | N | N | - | yes | 1 |
| **No 3** | P | N | N | N | *S. aureus* | no | 0 |
| **No 4** | N | N | N | N | - | no | 0 |
| **No 5** | N | N | N | N | - | yes | 2 |
| **No 6** | P | N | N | N | *S. aureus* | no | 0 |
| **No 7** | P | N | N | N | *S. agalactiae* | no | 0 |
| **No 8** | NA | N | N | N | - | no | 0 |
| **No 9** | P | N | N | N | *S. aureus* | no | 0 |
| **No 10** | P | N | N | N | *S. aureus* | no | 0 |
| **No 11** | P | N | N | N | *S. aureus* | yes | 2 |
| **No 12** | N | N | N | N | - | no | 0 |
| **No 13** | P | N | N | N | *S. pneumoniae* | no | 0 |
| **No 14** | *NA* | *NA* | N | N | - | yes | 2 |
| **No 15** | P | N | N | N | *S. aureus* | no | 0 |
| **No 16** | N | N | N | N | - | no | 0 |
| **No 17** | N | N | N | N | - | no | 0 |
| **No 18** | N | N | N | N | - | yes | 1 |
| **No 19** | N | N | N | N | - | no | 0 |
| **No 20** | P | N | N | N | *S. aureus* | yes | 5 |
| **No 21** | P | N | N | N | *Moraxella* | yes | 1 |
| **No 22** | N | N | N | N | - | yes | 1 |
| **No 23** | N | N | N | N | - | yes | 1 |
| **No 24** | N | N | N | N | - | no | 0 |
| **No 25** | N | N | N | N | - | no | 0 |
| **No 26** | P | N | N | N | *S. aureus* | no | 0 |
| **No 27** | N | N | N | N | - | yes | 2 |
| **No 28** | N | N | N | N | - | no | 0 |
| **No 29** | N | N | N | N | - | no | 0 |
| **No 30** | N | N | N | N | - | no | 0 |
| **No 31** | N | N | N | N | - | no | 0 |
| **No 32** | *NA* | *NA* | N | N | - | no | 0 |
| **No 33** | N | N | N | N | - | no | 0 |
| **No 34** | N | N | N | N | - | no | 0 |
| **No 35** | N | N | N | N | - | yes | 2 |
| **No 36** | *NA* | N | *NA* | *NA* | - | no | 0 |
